# The benefits, limitations and opportunities of preclinical models for neonatal drug development

**DOI:** 10.1242/dmm.049065

**Published:** 2022-04-25

**Authors:** Sarah Campion, Amy Inselman, Belinda Hayes, Costanza Casiraghi, David Joseph, Fabrizio Facchinetti, Fabrizio Salomone, Georg Schmitt, Julia Hui, Karen Davis-Bruno, Karen Van Malderen, LaRonda Morford, Luc De Schaepdrijver, Lutz Wiesner, Stephanie Kourula, Suna Seo, Susan Laffan, Vijay Urmaliya, Connie Chen

**Affiliations:** 1 Pfizer Worldwide Research, Development, and Medical, Groton, CT 06340, USA; 2 U.S. Food and Drug Administration, National Center for Toxicological Research, Division of Systems Biology, Jefferson, AR 72079, USA; 3 U.S. Food and Drug Administration, Center for Drug Evaluation and Research, Office of New Drugs, Silver Spring, MD 20993, USA; 4 Department of Experimental Pharmacology and Translational Science, Chiesi Farmaceutici S.p.A., 43122 Parma, Italy; 5 Pharma Research and Early Development, Roche Innovation Center Basel, Pharmaceutical Sciences, F. Hoffmann-La Roche, 4070 Basel, Switzerland; 6 Bristol Myers Squibb, Nonclinical Research and Development, Summit, NJ 07901, USA; 7 Federal Agency for Medicines and Health Products (FAMHP), Department DG PRE authorization, 1210 Brussels, Belgium; 8 Eli Lilly, Global Regulatory Affairs, Indianapolis, IN 46285, USA; 9 Janssen R&D, Preclinical Sciences & Translational Safety, 2340 Beerse, Belgium; 10 Federal Institute for Drugs and Medical Devices, Clinical Trials, 53175 Bonn, Germany; 11 Janssen R&D, Drug Metabolism & Pharmacokinetics, 2340 Beerse, Belgium; 12 GlaxoSmithKline, Non-Clinical Safety, Collegeville, PA 19406, USA; 13 Janssen R&D, Discovery Sciences, 2340 Beerse, Belgium; 14 Health and Environmental Sciences Institute, Washington, DC 20005, USA

**Keywords:** Drug development, Neonatal, Nonclinical

## Abstract

Increased research to improve preclinical models to inform the development of therapeutics for neonatal diseases is an area of great need. This article reviews five common neonatal diseases – bronchopulmonary dysplasia, retinopathy of prematurity, necrotizing enterocolitis, perinatal hypoxic–ischemic encephalopathy and neonatal sepsis – and the available *in vivo*, *in vitro* and *in silico* preclinical models for studying these diseases. Better understanding of the strengths and weaknesses of specialized neonatal disease models will help to improve their utility, may add to the understanding of the mode of action and efficacy of a therapeutic, and/or may improve the understanding of the disease pathology to aid in identification of new therapeutic targets. Although the diseases covered in this article are diverse and require specific approaches, several high-level, overarching key lessons can be learned by evaluating the strengths, weaknesses and gaps in the available models. This Review is intended to help guide current and future researchers toward successful development of therapeutics in these areas of high unmet medical need.

## Abstract (precis)

**Summary:** This article reviews and analyzes the available preclinical models for five common neonatal diseases to direct therapeutic development in these areas of high unmet medical need.

## Introduction

To some extent, pediatric patients remain therapeutic orphans (Smith and Davis, 2017). Neonates represent the most vulnerable therapeutic orphans, with the highest rate of off-label medicine use among the pediatric population. The reasons for this include the age-related challenges in clinical trials, small patient populations and unique disease conditions, as well as patients’ developmental heterogeneity, linked to their immature or premature status and inability for self-assessment. Hence, there is a need for model systems, including *in vivo*, *in vitro*/*ex vivo* and/or *in silico* ones, that can predict responses to therapeutics, and provide adequate safety assessments and dose or pharmacokinetic (PK) and pharmacodynamics (PD) predictions during medicine development for neonates.

The European Medicines Agency (EMA)’s Guideline on the investigation of medicinal products in term and preterm neonates (Table 1) came into effect in 2010 (https://www.ema.europa.eu/en/investigation-medicinal-products-term-preterm-neonate). Considerable experience has been gained in assessing pediatric investigation plans (PIPs) for neonates. Additionally, neonatal research trends and standards have come under debate in recent years, suggesting that the field is not adequately addressing the development and investigation of products used in this population. There is an ongoing effort to update currently available guidance for neonates (https://www.ema.europa.eu/en/documents/scientific-guideline/adopted-concept-paper-need-revision-guideline-investigation-medicinal-products-term-preterm-neonate_en.pdf; https://www.fda.gov/regulatory-information/search-fda-guidance-documents/general-clinical-pharmacology-considerations-neonatal-studies-drugs-and-biological-products-guidance). The specific recommendations within the proposal to revise the guidance include the development of more relevant models of neonatal disease, demonstration of the efficacy of therapeutics, and evaluation of short- and long-term safety. Toward this effort, the EMA reviewed 58 PIPs for neonates received between 2007 and 2015. A diversity of clinical indications was sought, including endocrine/metabolism, neurologic, cardiovascular, infectious disease, oncology and anesthesia/pain. Thirty-one of these PIPs included juvenile animal studies to support safety assessment. These were conducted in normal, healthy animals, thus, not designed to support a prospect of pharmaceutical benefit. The EMA identified 14 PIPs that included animal models of neonatal disease, such as bronchopulmonary dysplasia (BPD), specialized neurologic models and endocrine/metabolic models (Table 2). These specialized disease models were developed to provide proof of concept and were helpful in providing PK/PD correlations, as well as establishing a starting dose rationale. In addition to the animal models, *in vitro* models have also been utilized in the development of therapeutics for neonates. These include baby hamster kidney fibroblasts, adult and fetal tissue, cardiomyocytes, cultured cortical neurons, pediatric plasma and platelets, and cancer cell lines.

**Table 1.**
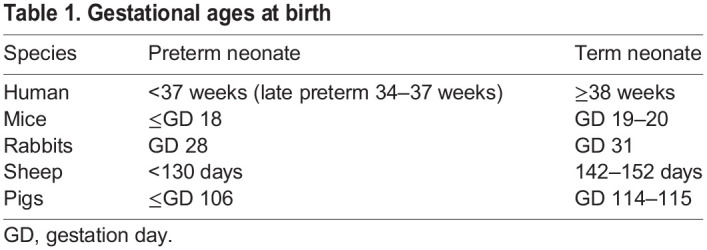
Gestational ages at birth

**Table 2.**
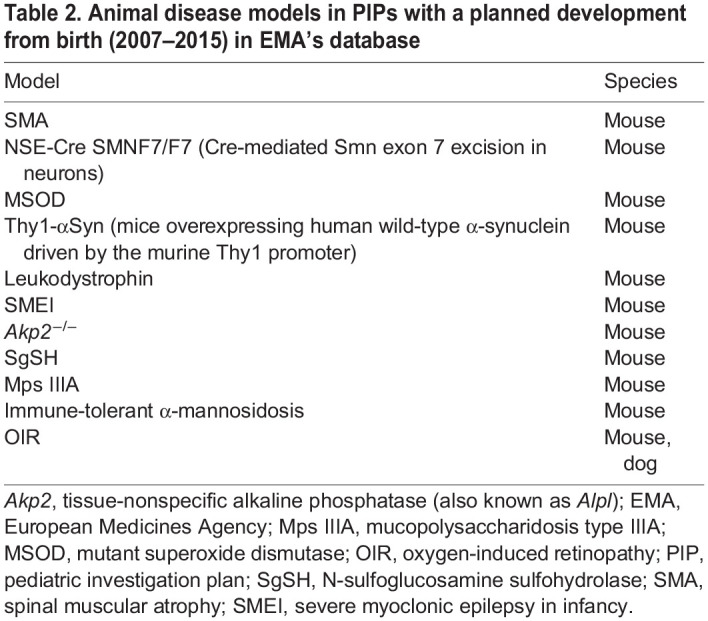
Animal disease models in PIPs with a planned development from birth (2007–2015) in EMA's database

Specialized neonatal disease models may add to the understanding of the mode of action and efficacy of a therapeutic and/or may improve the understanding of the disease pathology to aid in identification of new therapeutic targets. Here, we review five common neonatal diseases – BPD, retinopathy of prematurity (ROP), necrotizing enterocolitis (NEC), perinatal hypoxic–ischemic encephalopathy (HIE) and neonatal sepsis (NS) – and the available models for studying them. We provide an analysis of these models to direct therapeutic development in these areas of high unmet medical need. The five diseases of focus in this Review represent conditions for which effective models have been developed. However, it is our goal to provide insight into preclinical model development considerations that can be applied across a broad range of neonatal diseases.

## BPD

BPD is a chronic lung disease affecting preterm infants, arising as a consequence of preterm birth and neonatal intensive care management (Stoll et al., 2010). Its applied definition (Jobe and Bancalari, 2001) is based on diagnostic severity criteria, including oxygen need and an assessment of respiratory support. Despite a worldwide research effort, no BPD treatment has been approved to date (Poets and Lorenz, 2018). BPD is the result of a complex, interrelated and multifactorial process involving various pre- and postnatal factors that compromise normal lung development (Alvarez-Fuente et al., 2019), leading to impaired alveolarization often accompanied by variable degrees of inflammation, fibrosis and simplified vasculature (Nakanishi et al., 2016). The main BPD hallmarks can be replicated in a wide range of models, which were recently reviewed (Giusto et al., 2021).

### Animal models of BPD

**Fig. 1.**
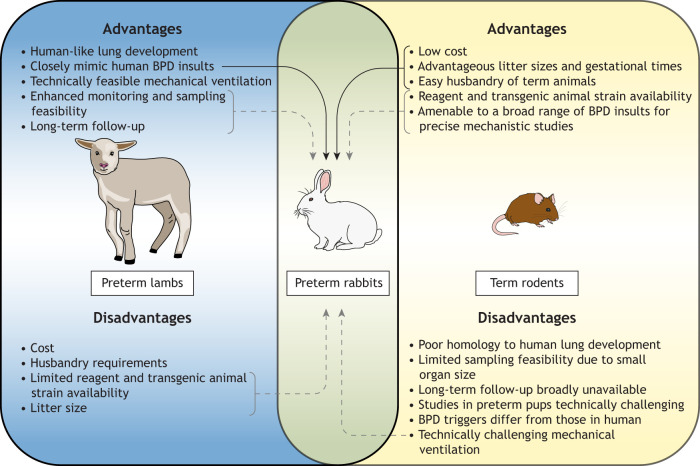
**Advantages and disadvantages of the preterm rabbit bronchopulmonary dysplasia (BPD) model in relation to the main other animal models, the preterm lamb and term rodents**. The rabbit model combines advantages (marked with solid line arrows) of large (lamb) and small animals (rodents) in recapitulating human BPD and, with further development, has the potential to overcome the remaining disadvantages (marked with dashed line arrows).

The most frequently used preclinical models of BPD can be divided into three classes: rodents born at term (Berger and Bhandari, 2014); preterm large-animal models, mostly lamb (O'Reilly and Thebaud, 2014) and non-human primates (NHPs) (Yoder and Coalson, 2014); and the preterm rabbit model (Fig. 1) (Berger and Bhandari, 2014; O'Reilly and Thebaud, 2014; Yoder and Coalson, 2014; Albertine, 2015; Salaets et al., 2017). These have been employed to investigate disease pathogenesis, identify new targets and test therapeutic approaches.

To induce the BPD phenotype, rodents have been exposed to both prenatal and postnatal insults, including lipopolysaccharide (LPS)-induced inflammation, intrauterine growth restriction, short-term mechanical ventilation, and exposure to different hyperoxia or hypoxia–hyperoxia cycles (Berger and Bhandari, 2014; O'Reilly and Thebaud, 2014; Nardiello et al., 2017). Additionally, a variety of transgenic mice have been developed to study the role of different genes in lung development and in its arrest. To our knowledge, models in premature mice or rats have rarely (Zhang et al., 2020) been utilized due to both logistic and lung development issues: term pups are managed by the doe, whereas preterm ones need intense care by operators; premature rodent pups do not match the preterm infant lung development stage. However, premature delivery and hyperoxia are the two main triggers of the BPD phenotype in the rabbit (Salaets et al., 2017). Additionally, pre- and postnatal inflammation, malnutrition and short-term mechanical ventilation could be potentially implemented to better model BPD in the preterm rabbit. Finally, lambs and baboons, owing to their bigger size, are the only models that allow closer mimicking of the main BPD triggers in humans: chorioamnionitis, premature delivery, intubation, application of both mechanical and less invasive ventilation, and exposure to oxygen levels similar to those employed in neonatal intensive care units (NICUs) [maximum fraction of inspired oxygen (FiO_2_) below 0.5] (Albertine, 2015; Allison et al., 2016; Hanita et al., 2017).

The recently developed preterm rabbit model is a suitable compromise (Fig. 1) between small- and large-animal models (Loi et al., 2021; Muhlfeld et al., 2021), sharing some of the advantages of rodents and lambs, such as short gestation, large litter size, and simple and inexpensive husbandry. Their intermediate size allows lung function measurements and intratracheal drug delivery, and feeding with a milk replacer allows the modeling of calorie restriction and malnutrition. Rabbit lung development is similar to human lung development, and researchers have the possibility to induce both functional and structural prematurity with preterm delivery while the lungs are in the early saccular stage of development (Salaets et al., 2017). The major disadvantages of rabbits are lack of reagents and the animal size, which is still too small for long-term ventilation and catheterization. Another important limitation of the preterm rabbit model is the inability to perform long-term experiments. Currently, experiments have been limited to 7 days due to potential issues of survival under 95% hyperoxia (Manzano et al., 2014). In contrast, both rodents and lambs allow researchers to follow the animal after the perinatal injury through adulthood to fully assess the long-term outcomes and effects of therapeutic interventions, including possible adverse effects. Although very informative, these long-term experiments are time consuming, expensive, and require years of care and protocol optimization (Dahl et al., 2018). The preterm rabbit remains a relatively new model and needs further optimization to render it suitable for long-term studies. Despite these limitations, an optimized rabbit model holds promise and could be the ideal system for initial therapeutic efficacy studies.

### *In vitro* models of BPD

*In vitro* and *ex vivo* BPD models have recently been developed to understand the processes of aberrant lung development (Giusto et al., 2021). These *in vitro* systems include organoid-like structures or 3D lung epithelial cell cultures in which different cellular components, as well as BPD-inducing insults, can be predefined and modified across experiments, allowing complete control of the system. Lung cells can be either derived from stem cells or purified and expanded from human fetal lung samples (Sucre et al., 2016, 2018a,b; Nikolic et al., 2017; Nikolic and Rawlins, 2017). A variety of exogenous insults like hyperoxia, hypoxia–hyperoxia cycles and the addition of TGF-β (also known as TGF-β1) have been employed to drive the BPD phenotype. The primary disadvantages of these systems are the absence of lung vasculature and immune cells, which are both central in lung development and BPD pathophysiology. Finally, *ex vivo* precision-cut lung slices are gaining popularity for alveolarization process studies (Davidovich et al., 2013; Akram et al., 2019; Sucre et al., 2020). These *in vitro* and *ex vivo* models could prove useful for understanding human alveologenesis, identifying new possible BPD targets, screening therapeutic compounds and performing preliminary efficacious dose studies.

### Assessment of preclinical models and clinical application

Although there is no approved treatment for BPD, some therapies that are now early-phase clinical trials have been tested and characterized in the models described above (Chang et al., 2014; Ahn et al., 2017; Pierro et al., 2017). One example is mesenchymal stem cell (MSC) therapy, for which extensive preclinical research has been carried out, mainly using the hyperoxia-exposed rodent model, in which MSCs and MSC-conditioned media improved the primary outcome of lung alveolarization and the secondary outcomes of pulmonary hypertension, lung inflammation, fibrosis and angiogenesis (reviewed in Mobius and Thebaud, 2016).

Clara cell 10 protein (CC10; also known as SCGB1A1) is a candidate therapeutic target that is abundant in the respiratory system, but deficient in premature infants. Although treatment with recombinant CC10 demonstrated acute efficacy in both lamb and rabbit models by reducing lung inflammation, improving pulmonary function, and upregulating surfactant protein and vascular endothelial growth factor (VEGF) expression (Miller et al., 2005; Wolfson et al., 2008), it did not reach the primary endpoint in a recently concluded clinical trial (Davis et al., 2019). In another example, preclinical studies in rodents indicated that restoring the insulin-like growth factor 1 (IGF-1) concentration may prevent or treat BPD by mitigating inflammatory lung injury and stimulating repair (Ley et al., 2013; Hellstrom et al., 2016; Seedorf et al., 2020), prompting the development of SHP607 (Shire Pharmaceuticals), composed of rhIGF-1 and its binding partner rhIGFBP3 (Ley et al., 2013). This molecule is now under investigation in a phase IIb clinical trial for the prevention of BPD (ClinicalTrials.gov Identifier NCT03253263). Finally, preterm lambs and preterm rabbits have been recently used to test the PK and efficacy of inhaled steroids (Roberts et al., 2016; Kothe et al., 2018, 2019; Gie et al., 2020; Hillman et al., 2020a,b). These preclinical studies are being performed in concert with clinical studies to complement and guide the design of new clinical trials for postnatal anti-inflammatory therapies (Yeh et al., 2008, 2016; Bassler et al., 2015).

The candidate BPD therapies described above demonstrate the utility of currently available BPD models in the clinical development of new BPD drug candidates. Despite continuous refinement, several important questions are still outstanding (Morty, 2020). First, it is not completely clear how well these models recapitulate BPD. The majority of available tissue samples from patients date to when the BPD phenotype was mainly caused by ventilatory injury and not extreme prematurity, like it most often occurs today. Some of the main phenotypes in animal models are alveolar simplification, septal thickening and arrested vascular development. However, it is unclear how well these mimic human BPD, because the exact nature of capillary and alveolar septa perturbation are largely unknown due to the paucity of ‘new’ BPD histological samples. Moreover, it is important to appreciate that BPD is a syndrome that can present with variable and mixed features that vary from patient to patient and in different gestational ages at birth (Morty, 2018). Improving the characterization of the BPD phenotype in patients will aid the development of appropriate preclinical models and their use. In summary, there is a discordance between the currently available preclinical models and the clinical presentation, with several studies demonstrating that a beneficial impact of a new therapeutic approach can be masked if the initial phenotype-inducing insult is too severe (Nold et al., 2013; Nardiello et al., 2017; Rudloff et al., 2017). A clearer understanding of the clinical BPD phenotype and more refined preclinical models will aid in avoiding these issues and improve successful translation of candidate therapies into the clinic.

## ROP

ROP is an eye disease characterized by abnormal neovascularization of the retina. Each year, 14,000–16,000 premature infants in the USA that are born before 31 weeks of gestation and weigh less than 1250 g develop ROP (National Eye Institute, 2019). Fortunately, 90% of cases are classified as mild and do not require treatment. However, 1100–1500 infants develop advanced ROP requiring invasive medical interventions, e.g. photocoagulation, cryopexy, scleral buckling or vitrectomy. Clinical trials with antioxidants like vitamin E and D-penicillamine, recombinant human growth factors, VEGF inhibitors and dietary supplements like omega-3 polyunsaturated fatty acids have also been explored as possible therapies (Aranda et al., 2019; Hartnett and Toth, 2019). Even with treatment, ∼400–600 children diagnosed with ROP in the USA become legally blind each year. Both animal and human studies have identified genetic susceptibilities that increase the risks for ROP development and its progression (Bizzarro et al., 2006; Holmstrom et al., 2007).

The development of ROP in premature infants has been associated with varying levels of supplemental oxygenation (Campbell, 1951), with further studies revealing a biphasic progression. It is thought that giving a premature infant supplemental oxygen inhibits the production of VEGF, resulting in an incomplete vascularization of the retinal periphery. This leads to retinal hypoxia once the infant is removed from supplemental oxygen, and the condition is exacerbated by the further development of the retina and its continuing demand for oxygen. This retinal hypoxia stimulates a ‘second wave’ of vascular development, which is characterized by neovascularization from the retina into the vitreous cavity. These abnormal vessels are fragile, resulting in hemorrhage and scars that may ultimately lead to retinal detachment and blindness. Animal data also suggest that systemic inflammation is one of the pathogenic mechanisms and that short periods of systemic acidosis may be an additional independent ROP risk factor (Yanni, 2010).

### Preclinical models of ROP

The term oxygen-induced retinopathy (OIR) describes many of the animal models approximating ROP in infants. Previous publications have extensively reviewed the mouse, rat, cat and dog models of OIR, describing their contributions and limitations for understanding and treating ROP (Madan and Penn, 2003; Yanni, 2010). Those ‘classic’ OIR models are not reviewed here, as we instead focus on newer models such as zebrafish, rat LPS and rat systemic acidosis (AIR) models.

The mouse is a popular model for the study of ROP (Scott and Fruttiger, 2010) and has provided valuable insight into the underlying genetic factors and regulators. However, the eyes of a mouse pup do not open until around postnatal day (P)14; therefore, the eyes are largely not visible or easily manipulated during critical periods of ROP development. The vascularization of a newborn rat resembles that of a premature infant, with retinal vascularization completed by P15. The OIR rat model is created by exposing newborn pigmented or albino rats to cycling conditions of 50% and 10% oxygen every 24 h for 14 days. The cycling oxygen profile more closely reflects fluctuating lung function and subsequent changes in arterial blood oxygen of an infant in the NICU and can induce retinopathy in 97% of the pups. The angiogenic pattern mimics that of ROP. Thus, the OIR rat is thought to be a relevant model to the human condition. When hypoxia is limited, lasting minutes rather than hours and in clustered episodes, a more severe form of OIR develops compared to rats undergoing dispersed hours-long hypoxia (Coleman et al., 2008).

The rat LPS model uses systemic inflammation to alter retinal vascular development. Intraperitoneal injection of LPS at P1, P3 and P5 suppresses retinal vascularization and rearranges the retinal vascular structures (Hong et al., 2014). This combines with abnormal vascular tuft formation and peripheral ridge formation to resemble ROP. Retinal capillary obliteration and neovascularization are less prominent in the LPS compared to the OIR model. However, the LPS model develops a ridge formation in the peripheral vascular boundary that recapitulates an essential step in severe ROP in humans. Increased apoptosis in the retina and decreased thickness of the inner retina have also been noted in the LPS model.

There are several rat models of retinopathy induced via systemic acidosis, e.g. resulting from CO_2_ inhalation (Holmes et al., 1998, 1999), NH_4_Cl gavage (Chen et al., 2002) or acetazolamide injection (Zhang et al., 2001). Even systemic metabolic acidosis periods as brief as 24 h induce neovascularization in the neonatal rat similar to that of ROP. The ratio of vascularized-to-total retinal area is smaller in AIR than in controls. This suggests that short periods of systemic acidosis, similar to those observed in critically ill neonates, may be an important and independent risk factor for ROP.

The porcine eye, including the minipig, shares many anatomical and physiological similarities with the human eye. Göttingen minipigs have become a popular non-rodent animal model for ocular research (Shrader and Greentree, 2018); long-standing practical experience with pig models allows for perinatal manipulation, including work with preterm newborns (Vrolyk et al., 2020). Piglets can be kept alive over several days in incubators with ventilator support and oxygen, blood gas and electrolyte monitoring. A piglet ROP model using cesarean section around day 100 of gestation (full term by 114 days; Table 1), comparable to 35 weeks of gestation in humans, applies hyperoxia and hypoxia during a period of ∼1 week (Ifantides et al., 2014). This model exhibits similar characteristics to humans with ROP, as well as to other proliferative retinopathy animal models. Pig models are considered more advantageous due to these practical and scientific advantages.

Limited studies have used the rabbit for investigating ROP (Myers et al., 2012). This is, in part, because the retinal vessels in the rabbit are limited to a small region and extend horizontally on either side of the optic disk. The unique feature of the rabbit is retinal neuron damage when exposed to hyperoxia, which is not observed in other models. However, retinal detachment does not occur in the rabbit OIR model (Madan and Penn, 2003).

The zebrafish provides a comparably simple and quick *in vivo* model for the evaluation of effects and treatments, while allowing large sample sizes and statistical power. A green fluorescent vascular endothelium zebrafish transgenic line, Tg(*fli1a:EGFP*), has been used to study ROP. Embryos at 1 day postfertilization are treated with cobalt chloride to induce hypoxia and subsequently with GS4012, a VEGF inducer, to stimulate neovascularization. The number of vascular branches and sprouts significantly increases in the central retina vascular trunks 2–4 days after treatment (Wu et al., 2015).

### *In vitro* and *in silico* models of ROP

Limited *in vitro* models have been reported for ROP. Initial studies were conducted on the intact vascular complex from rabbit eyes (Tripathi et al., 1973). Exposure to high levels of oxygen caused shrinkage and retraction of the capillaries, with the return to ‘room air’ stimulating capillary growth. In a hyperoxia/hypoxia *in vitro* cell model based on human umbilical vein endothelial cells (HUVECs), cell proliferation and migration were compromised, consistent with the inhibition of angiogenesis in early-stage ROP and, therefore, showed potential in mimicking ROP vascular injury (Zhu et al., 2017). However, it is difficult to extrapolate the relevance of *in vitro* oxygen exposure to physiologic conditions and of the *in vitro* findings to the pathogenesis of ROP, limiting the translational potential of these models. More recently, primary human mixed retinal cultures have shown promise for evaluating changes in the retina and identifying potential drug targets (Shahulhameed et al., 2019).

A continuum mathematical model consisting of systems of partial differential equations (PDEs) was used to develop an *in silico* representation of the developing murine retinal vasculature (Aubert et al., 2011). The ‘basic’ model described the migration of endothelial tip cells, the migratory response of the blood vessels and the effect of VEGF concentration. An extension of the model incorporated the migratory response of astrocytes to PDGF gradients and VEGF secretion by hypoxic astrocytes. Overall, the models described by Aubert et al. (2011) accounted for the evolution of capillary blood vessel density and growth factor concentration to model the formation of the retinal vascular plexus. Early simulations indicated an excellent correlation with *in vivo* data, but the model remains inadequate, particularly due to its simplification in VEGF production, which does not fully reflect the biological profile in the developing retina.

Subsequent work led to the development of a more complete hybrid PDE discrete model to track the migration of endothelial tip cells and astrocytes in response to biological cues (McDougall et al., 2012). The *in silico* retinal vascular plexus matched closely with the *in vivo* whole mounts at various stages of development. Upon showing good correlation with the wild-type *in vivo* data, the model was used to predict the effect of overexpression of various transgenes on VEGF-A expression. This type of information could inform future predictions of aberrant angiogenesis in the context of ROP (Watson et al., 2012).

### Assessment of preclinical models and clinical application

In general, no existing preclinical model fully mimics ROP in the human premature neonate (reviewed in Madan and Penn, 2003; Yanni, 2010). Unlike the premature infant, the model animals are generally healthy and born at a normal developmental stages, except for the piglet. Additionally, animal models generally use extreme oxygen concentrations and postnatal/lactational exposure protocols, which are not necessarily relevant to conditions and exposure windows in the clinic. Nevertheless, critical structural hallmarks of ROP can be induced in these models, which allow mechanistic investigation and the testing of potential therapies. One possible confounding factor in all models of ROP is that growth retardation appears to correlate with the neovascularization.

Rodent models of OIR have several scientific drawbacks. In mouse, the central retina is avascular and the peripheral retina is vascularized, which is opposite to the situation in humans with ROP, in whom the peripheral retina remains avascular and the central retina is vascularized. Additionally, retinal detachment as a clinical hallmark of ROP is not observed in mice. One possible confounding factor in all models of ROP is that growth retardation appears to correlate with neovascularization. The rat is generally considered the best characterized model of OIR among all model animal species.

The cat model, aside from the higher costs associated with maintaining cats in a research setting, also has drawbacks. The vasoproliferation pattern in cats is different from that observed in human ROP patients, even though intraretinal and intravitreal neovasculogenesis is observed. The cat does, however, show iris vascular engorgement, pupillary rigidity and anterior segment abnormalities like infants with ROP. The cat model does not develop retinal detachment, and the observed neovascularization regresses with time. The beagle pup has been suggested as a superior model to study ROP because it is the only established model in which a high percentage of animals develop retinal neovascularization with subsequent retinal detachment (Flower et al., 1985), and retinal folds and detachments have been found to spontaneously occur in dogs. The remaining gaps in a more human-relevant model (i.e. preterm condition) could be filled by the pig, as this species allows for manipulations such as use of ventilator support and monitoring of oxygen, blood gas and electrolytes, and the size and morphology of the eye closely resembles that of humans. Given these advantages, more research in pig/minipig models is highly encouraged.

The consistent production of human-like patterns of vaso-attenuation and vaso-proliferation observed in the OIR rat has made it a popular small-animal model. The rat has been used to test the efficacy of anti-angiogenic compounds, as well as drug delivery routes. In addition to the OIR rodent models, the cat and dog models have been used to support clinical trial development and investigate potential therapeutic benefits. Specifically, the Supplemental Therapeutic Oxygen Protocol for Retinopathy of Prematurity clinical trial (ClinicalTrials.gov Identifier NCT01203436) was developed, in part, based on experiments in cats, while the OIR dog model was used to test the potential therapeutic benefit from VEGF receptor antagonists and monoclonal RhoB antibody treatment (Madan and Penn, 2003; Almonte-Baldonado et al., 2019).

## NEC

NEC is one of the most severe disorders occurring in NICUs, with a prevalence ranging from 3% to 10% across the USA (Alsaied et al., 2020) and an estimated incidence of one to three cases per 1000 live births (Fox and Godavitarne, 2012; Stoll et al., 2015). NEC incidence is inversely correlated to gestational age and birth weight (Sharma et al., 2006; Markel et al., 2014). NEC can develop extremely rapidly from an initial inflammatory response to intestinal necrosis and perforation, leading to sepsis and multi-organ failure, which makes it the most common cause of death between 15 and 60 days of life in very-low-birth weight (VLBW) infants (Stoll et al., 2010; Neu and Walker, 2011). Although so-called full-term or late-preterm NEC occur almost exclusively in infants with intestinal anomalies, a hypoxic–ischemic insult, or cyanotic congenital heart disease (Caplan et al., 2019), NEC can also occur in preterm infants, in whom less is known regarding the specific pathogenesis. Owing to its unknown pathogenesis, treatment strategies are limited to bowel rest, antibiotics, total parenteral nutrition and surgical intervention in severe cases. No validated biomarkers for early disease onset have been identified, and, often, only pneumatosis intestinalis or intestinal perforation accompanying advanced systemic illness ensures the diagnosis (Neu and Walker, 2011; Tiwari et al., 2015). NEC-associated mortality has remained between 20% and 30% over the past 50 years, reaching 50–65% in surgical cases. Long-term sequelae like short bowel syndrome, poor growth and neurodevelopmental impairments are frequent among survivors (Neu and Walker, 2011; Tanner et al., 2015; Cho et al., 2016).

A clearer understanding of the molecular mechanisms underlying NEC pathogenesis is needed to identify biomarkers for early recognition of disease onset and targetable molecules for effective intervention. Toll-like receptor (TLR) 4 activation (Jilling et al., 2006), TNF-α (also known as TNF) stimulation (Halpern et al., 2006), NF-kB signaling (De Plaen et al., 2007), increased platelet-activating factor levels (Caplan et al., 1997; Frost et al., 2008) and decreased EGF (Dvorak et al., 2002) have been identified as key players in NEC pathogenesis. Subsequent studies have focused on bacterial dysbiosis (Wang et al., 2009; Torrazza et al., 2013), as well as the roles of the innate (MohanKumar et al., 2012) and adaptive immune systems (Weitkamp et al., 2013) as main contributing factors to the development of NEC. Recent studies investigated serum citrulline levels as a potential biomarker (Feenstra et al., 2021; Jawale et al., 2021). These are promising, but require further validation in preclinical models and in the clinic.

### Animal models of NEC

Several features of NEC complicate the development of a robust preclinical model. All models are based on one or more consistently occurring risk factors of NEC in humans: prematurity, enteral feeding, bacterial colonization and intestinal ischemia (Kovler et al., 2020). But the fact that NEC develops in premature babies several weeks after birth exacerbates the challenge. Mimicking this condition in neonatal mice is extremely difficult given their small size and weight. Moreover, there is no known specific genetic variant that would lead to spontaneous NEC development (Sodhi et al., 2008). Finally, because NEC demonstrates a complex pathophysiology involving prematurity of the intestine and immune system, bacterial colonization and potentially some genetic predisposition or other triggers, capturing all factors in one relevant animal model is a major challenge. Although each existing model has its specific drawbacks, there are common limitations including the lack of genetic diversity in laboratory animals, static environmental conditions and innate immune systems, and intestinal development trajectories that differ from those of humans (Sangild, 2006; Tanner et al., 2015).

One of the earliest NEC models, a neonatal rat, employed enteral feeding, *Klebsiella* and hypoxia (Barlow et al., 1974). It has been refined over the years (Caplan et al., 1994; Halpern et al., 2006; De Plaen et al., 2007), often using slightly modified protocols. Rat pups are usually delivered by cesarean section, stabilized and then exposed to continuous enteral feeding either via a gastric pump (Jilling et al., 2004) or oral gavage (Dvorak et al., 2002, 2003) over 3–4 days, in addition to asphyxia (Caplan et al., 1994, 1997) or hypoxia (Dvorak et al., 2002, 2003), and/or cold stress and bacteria in some cases. This model, generally referred to as the hypoxia/gavage (H/G) model, consistently leads to epithelial cell sloughing, villus damage and destruction, severe submucosal edema, separation of the mucosa and lamina propria, and intestinal necrosis in the most severe cases (Tanner et al., 2015). Overall, ∼60–80% of the rats develop moderate to severe histopathology at the distal ileum and proximal colon (Caplan et al., 1994; Dvorak et al., 2002; Halpern et al., 2002; Jilling et al., 2004), similar to human NEC. Clinical signs of NEC, like abdominal distention, delayed gastric emptying, bloody stools and respiratory distress, have also been described (Caplan et al., 1994; Dvorak et al., 2003; Halpern et al., 2008). The H/G model thus represents several hallmarks of human NEC. The use of animals delivered by cesarean section includes prematurity and excludes the exposure to maternal milk. However, the complexity and duration of the modeling process challenge data reproducibility and require extensive planning and technically trained investigators. Moreover, the model uses premature pups immediately after cesarean delivery, whereas in humans, NEC only presents 1–2 weeks after birth, and the more premature the neonate, the later the onset (Gonzalez-Rivera et al., 2011). Therefore, the H/G model does not fully recapitulate the human condition. Additionally, the model relies heavily on hypoxic injury, but the involvement of hypoxic ischemic injury in NEC is under debate (Neu, 2014).

Several mouse models of NEC exist and have been reviewed by Lu et al. (2014) and Tanner et al. (2015). The original mouse model applied the H/G protocol to neonatal pups delivered by cesarean section (Jilling et al., 2006) or immediately after birth (Halpern et al., 2008). However, owing to technical challenges, most investigators employ 7- to 10-day-old mouse pups, often justified by the argument that rodents have a very immature gastrointestinal tract (GIT) at birth. In contrast, the maturation of the human intestine starts *in utero*, such that digestion of non-milk carbohydrates and proteins is already sufficiently functioning at term birth (Sangild, 2006). Although scientific evidence has been collected in ontogeny studies (Lebenthal and Lebenthal, 1999; Montgomery et al., 1999; Zabielski et al., 1999; Sangild, 2006), the exact comparability of the rodent and human intestine at certain developmental stages is still under investigation. Using neonatal mice between 1 and 2 weeks of life removes or reduces aspects of intestinal prematurity and allows exposure to dam milk until the start of the experiment. The main characteristics, as well as the pros and cons of murine models of NEC, are summarized in Table 3. It should also be noted that these models can be subject to strain differences. Therefore, different mouse strains may require varying intensity of intervention to induce NEC.

**Table 3.**
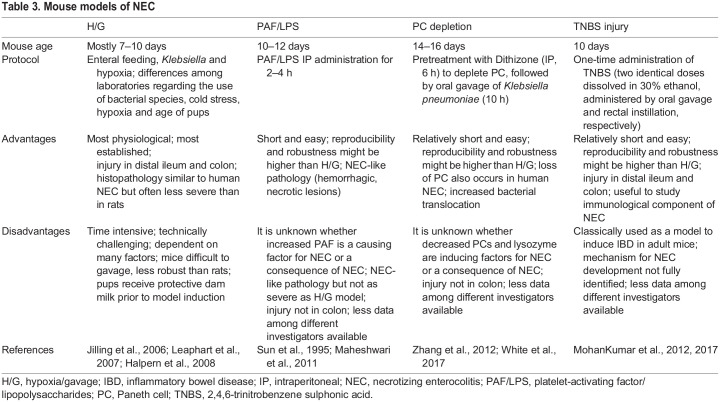
Mouse models of NEC

The pig is an attractive animal model for NEC because the development, physiology and functionality of its GIT most closely resembles that of humans, especially in the neonatal and newborn stages (Sangild et al., 2013). In neonatal term pigs, NEC is induced with similar approaches to the H/G rat, using different combinations of hypoxia (Cassutto et al., 1989), hypothermia (Cohen et al., 1991) and/or formula feeding (Crissinger et al., 1994). Because piglets are very susceptible to NEC, the insults are not necessarily applied together, and the duration required for NEC development can be shorter than in rats. The preterm pig model recapitulates human NEC. Interestingly, preterm pigs delivered before 95% of gestation suffer from complications related to the same immaturities as preterm infants, like impaired respiratory, nutritional, immunological and metabolic responses (Sangild et al., 2013). Another advantage of the preterm pig model is its similarity in size to preterm human neonates. Prematurely delivered pigs (Table 1) develop NEC spontaneously with an incidence between 30% and 90%, which is higher than in humans, if kept under enteral feeding with formula instead of colostrum and if large feeding volumes are applied. The signs and histopathology demonstrate striking similarities with human NEC (Sangild et al., 2013). In fact, the pig is the only animal model that develops the two most important hallmarks of NEC, spontaneous development and pneumatosis intestinalis. Because NEC in this model only depends on enteral feeding and prematurity, it may be more robust than rodents, which require several additional triggers. However, the pig model requires a surgical facility, a piglet intensive care unit (ICU) and trained personnel, and is therefore cost and labor intense.

### *In vitro* models of NEC

The small intestinal epithelium has a high self-renewing rate with a turnover of less than 5 days. In 2009, Hans Clevers' group developed long-term organoid cultures of the intestinal epithelium from intestinal stem cells (Sato et al., 2009). Shortly after, organoid culturing from human small intestinal and colonic biopsies was described (Sato et al., 2011). Hartman et al. (2013) and Senger et al. (2018) described the use of fetal-derived organoids as a potential human NEC model and demonstrated that treatment with LPS and *Escherichia coli* elicited an innate immune response in these organoids. Further developments of intestinal organoids derived from premature babies and co-culture systems with immune cells and the microbiota are already on the way (Kovler et al., 2020). Several investigators have recently demonstrated that intestinal organoids derived from juvenile mice can be employed as an *in vitro* NEC model to gain further mechanistic insights (Li et al., 2019; Werts et al., 2020). Applying stressors like hypoxia and bacteria (or LPS) to murine intestinal organoids resulted in a NEC-like *ex vivo* culture with epithelial cytokine response, tight junction impairment, tissue architecture disruption and cell death. Using this *ex vivo* NEC model also enables studies with mice from different genetic backgrounds.

Intestinal organoids are thus perfectly suited to close the gap between the not-fully representative animal models and human NEC. Intestinal organoids derived from either NEC patients and age-matched controls (Dedhia et al., 2016; Spence, 2018) or from juvenile animals and exposed to NEC stressors holds great potential to identify NEC pathogenesis mechanisms and for target screening, drug toxicity evaluation and metabolism studies. However, like in animal models, clear endogenous biomarkers confirming NEC in these *in vitro* models are necessary to validate their translational value. Additionally, unless co-cultured with immune cells, organoids only account for the epithelium and cannot yet capture the overacting immunity that amplifies epithelial damage in NEC *in vivo* Kasendra et al., 2018).

### Assessment of preclinical models and clinical application

Despite recent research efforts, the pathogenesis of NEC is still not fully characterized. Thus, treatment strategies are limited and non-specific. None of the described models have been applied for drug development to date. Different investigators have demonstrated that EGF (Dvorak et al., 2002), heparin-binding EGF (Feng et al., 2006), anti-TNF (Halpern et al., 2006) and probiotics (Khailova et al., 2009) significantly attenuate NEC severity in the rat model. Owing to its establishment and the frequent use of rats in pharmaceutical drug development, the H/G rat could be applicable for target identification, as well as for safety and efficacy studies. To ensure this, investigators should establish consistent experimental conditions to overcome the irreproducibility associated with this model.

To date, the preterm pig model has mainly been used to improve parenteral and enteral feeding protocols. However, it holds great potential for target identification, safety and proof-of-concept studies. Moreover, among all the animal models presented above, it most closely resembles the symptoms, histopathology and spontaneous development of NEC in humans. Drawbacks for large-scale testing are the cost-intense sow surgical facilities and piglet ICUs, and trained staff requirements. For specific pathway identification, to gain better understanding of the pathogenesis or to focus on the immunological aspect of NEC, *in vitro* or small-animal models might be the better choice.

Each of the models described exhibit strengths and drawbacks. An ideal NEC model should reflect the distinct GIT and immune system developmental situation in premature babies. Challenges for modeling are the interplay between impaired epithelium and overactive immune system in the premature setting. The ideal model also needs robustness regarding incidence, injury and readouts. Finally, identifying biomarkers for NEC would enable an investigator to perform unbiased studies independent from subjective histopathology scoring.

## Perinatal HIE

HIE in the late-gestation/term infant is by far the most common cause of neonatal brain injury and a major cause of acute mortality and chronic neurological morbidity in infants and children (Nair and Kumar, 2018). HIE affects 2–4/1000 full-term births and is frequently associated with neurocognitive impairment, cerebral palsy and seizure disorders (Novak et al., 2018). Therapeutic hypothermia has successfully reduced neurologic disability in survivors of moderate to severe HIE, and has thus been adopted as standard of care for neonates born at at least 36 weeks' gestation (Jacobs et al., 2013). Current hypothermia protocols have consistently involved starting treatment within the first 6 h of life, with 34.5±0.5°C for head cooling, or 33.5±0.5°C for whole-body cooling, and continuing treatment for 48–72 h (Davidson et al., 2015). Novel therapeutic strategies that aim to augment neuroprotection and/or neuroregeneration in combination with cooling, and thus reduce the number of affected infants, are highly needed (Nair and Kumar, 2018). Relevant preclinical models continue to be essential to elucidate mechanisms and to develop neuroprotective strategies.

HIE of sufficient severity to deplete cerebral energy reserves activates multiple pathways, including disruption of calcium homeostasis, microglial activation, excitotoxicity, and oxidative and nitrosative stress, leading to ongoing cell death (Yildiz et al., 2017). Although the early period of reperfusion partially restores cerebral energy stores, numerous studies in both asphyxiated infants and preclinical animal models have demonstrated a secondary depletion of cerebral high-energy compounds, or secondary energy failure, during the subsequent hours to days (Patel et al., 2014), often characterized by neuroinflammation (Borjini et al., 2019) and further cellular damage (Hagberg et al., 2015).

### Preclinical models of HIE and brain injury

#### Term and juvenile animal models

Commonly used experimental models of neonatal encephalopathy involve induction of hypoxia–ischemia (HI) or focal ischemic lesions in juvenile rodents, with or without the addition of inflammation (Patel et al., 2014). The discovery that mild, induced hypothermia can improve neurological recovery after moderate to severe HI was initially demonstrated in rodent and piglet preclinical models (Gunn and Thoresen, 2015). Because therapeutic hypothermia is already a routine standard of care for neonatal encephalopathy, it is important that this therapy is utilized in the selected experimental model to benchmark novel therapeutic strategies (Sabir et al., 2014). Moreover, a translational approach requires that therapeutic interventions must be initiated after reperfusion and tested also in combination with therapeutic hypothermia (Robertson et al., 2019).

**Fig. 2.**
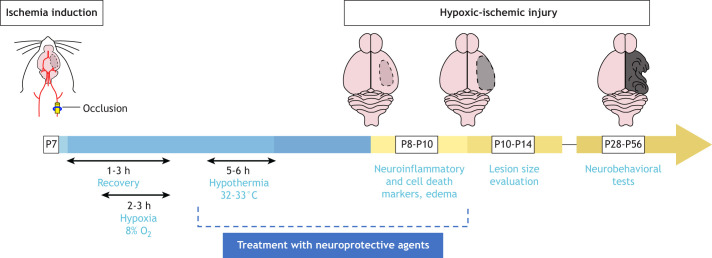
**Experimental design and common timelines and readouts of the classic Rice–Vannucci hypoxia-ischemia (HI) model of perinatal hypoxic–ischemic encephalopathy.** In this model of neonatal stroke, 7-day-old rat pups undergo unilateral occlusion of the common carotid artery to cause a brain injury. Such occlusion can be transient or permanent and can be obtained through ligation, electrocauterization or guided magnetic nanoparticles, followed by exposure to 8% oxygen hypoxic air. Modifications of oxygen saturation, age at injury, duration of hypoxia, and reoxygenation have been reported in the literature (Vannucci and Vannucci, 2005). Cerebral hemisphere damage ipsilateral to the carotid artery occlusion occurs upon HI and is associated with neuroinflammation, edema, and motor and cognitive deficits. Inflammation typically arises 24–48 h postinjury and neurological deficits are typically assessed postweaning. This model allows researchers to test novel neuroprotective treatments, and, indeed, this model was essential in establishing neuroprotective hyperthermia as a clinical standard of care. Figure adapted from Rumajogee et al. (2016) and Borjini et al. (2019) under the terms of the CC-BY 4.0 license. P, postnatal day.

The classic Rice–Vannucci HI model, in which 7-day-old rat pups undergo unilateral ligation of the common carotid artery followed by exposure to 8% oxygen hypoxic air, is a model of neonatal stroke that has greatly contributed to research in this area (Taniguchi and Andreasson, 2008). Modifications of the method, i.e. oxygen saturation, age at injury, duration of hypoxia and reoxygenation, have been described (Vannucci and Vannucci, 2005). The model has also been adapted to genetically modified mice for mechanistic studies and target validation (Klofers et al., 2017) (Fig. 2). Cerebral hemisphere damage ipsilateral to the carotid artery occlusion upon HI is associated with neuroinflammation and motor and cognitive deficits (Rumajogee et al., 2016). The Rice–Vannucci HI model is well developed and widely applied to study hypothermia as a neuroprotective strategy in combination with other agents (Patel et al., 2014; Sabir et al., 2012). To date, most rodent studies collect data upon 5–6 h of therapeutic hypothermia at 32–33°C. Rodent models of neonatal focal ischemia are obtained by occlusion of the middle cerebral artery (reviewed in Hamdy et al., 2020). By allowing reperfusion, transient occlusion provides a more clinically relevant model of neonatal stroke in humans (Tsuji et al., 2013), although this limits its applicability to the variable conditions associated with neonatal HI insults, which are more extensively recapitulated by the Rice–Vannucci HI model. Limited oxygen delivery to the neonatal brain can occur for several reasons beyond ischemic events. Hypoxic models, including acute, chronic continuous and chronic intermittent hypoxia, are also used for neonatal brain injury studies (Kanaan et al., 2006; Schneider et al., 2012).

Numerous rodent models are available to study neonatal hypoxia, ischemia and HI, a detailed description of which is beyond the scope of this Review. The rodent Rice–Vannucci HI model and its adaptations remain the most convenient, cost-effective and widely used animal model for recapitulating neonatal HIE and for testing new therapeutic interventions (Patel et al., 2014). These are also useful for studying mechanisms, biological pathways and responses to therapeutic hypothermia, thus allowing translational testing of drugs in combination with current standards of care. Following neurological functional parameters and/or lesion evolution over time is also possible with neuroimaging and other readouts (Back et al., 2012). Additionally, rodent pups do not require intensive care after induction of a brain lesion.

Limitations of rodent models include the major differences between rodent (lissencephalic) and human (gyrencephalic) brain structure and development. Uniform HI insults can result in great variability in infarct volumes not only between rodent strains but also among littermates. The relatively large lesions observed in the rodent HIE model do not always translate to severe neurological deficits, as would likely in humans (Rumajogee et al., 2016). Additionally, rodent responsiveness to neuroprotective treatments is highly dependent on the extent of the HI-induced brain lesion (Sabir et al., 2012) and can be sex dependent (Klofers et al., 2017).

#### Piglet model of neonatal encephalopathy

Brain structure and development in a newborn piglet are more similar to those in human than to those in rodents. The peak brain growth spurt in piglets and humans occurs near the time of birth, whereas it is postnatal in rodents. A severe hypoxia model in which anesthetized newborn piglets are orally intubated and mechanically ventilated with 6% O_2_ for as long as 45 min has been described (Thoresen et al., 1996). More recently, a systemic hypoxia model has been reported (Robertson et al., 2019). Although newborn piglets undergo partial restoration of reperfusion, a secondary depletion of cerebral high-energy compounds or secondary energy failure occurs during the subsequent hours to days, indicating lasting brain injury (Fleiss et al., 2015). Importantly, newborn piglets provide the opportunity to study longer periods of therapeutic hypothermia than rodents. The piglet is amenable to intravenous drug infusions alongside hypothermia (Fleiss et al., 2015). The piglet body surface area and cardiovascular physiology are comparable to those of human neonates, allowing more accurate PK and human dose predictions (Robertson et al., 2019). Real-time monitoring of physiological parameters also allows safety studies (Robertson et al., 2019). Translational neuroimaging readouts showed that the model mimics many aspects of human HIE (Mitra et al., 2018; Pang et al., 2020).

Experiments in the piglet model can only be conducted up to 48–72 h postlesion, which precludes follow-up on neurological and functional parameters and/or lesion evolution over time. Piglet studies require an ICU and trained personnel, which is associated with high costs. Because differences between protocols result in variable extents of brain injury, a real-time titration of the HI insult and recovery using cerebral biomarkers is recommended to reduce variability (Robertson et al., 2014).

#### Near-term large-animal models

Different NHP models of HIE encompass partial or complete asphyxia at the time of delivery. A near-term birth asphyxia model has been developed in *Macaca nemestrina* by transiently occluding the umbilical cord before birth (Traudt et al., 2013). The animals develop severe asphyxia associated with cerebral palsy-like motor abnormalities (Traudt et al., 2013). Generally, in NHP, complete asphyxia preferentially injures the cerebellum and primary sensory nuclei in the brainstem and thalamus, whereas partial asphyxia injures the somatosensory, motor cortex and subcortical nuclei (Koehler et al., 2018). However, costs, ethical issues and the complexity of setting up a NHP facility limit the availability of this model for drug discovery. There is a desire to minimize the use of NHP to only essential circumstances.

Sheep studies are performed during pregnancy to correlate with relevant maturation stages in humans (Drury et al., 2013). Cerebral ischemia models in fetal sheep, induced by bilateral transient occlusion of the carotid arteries, were first developed in the near-term fetus and, later, during mid-gestation. Such models can also involve therapeutic hypothermia (Davidson et al., 2016). The chronic fetal sheep umbilical cord occlusion model is globally used and allows examination of intrauterine pathophysiology and the contribution of other organs to HIE without the influence of anesthesia (Back et al., 2012). However, fetal models are complicated by maternal/placental metabolism, which is not present in the human term HIE. In preterm sheep models, fetuses are more prone to white and deep-gray matter injury, and the vulnerability of cortical gray matter increases with advancing gestation.

In rabbits, intrauterine ischemia is induced at 92% gestation to mimic at-term injury. Imaging of deep brain injury 6–24 h after near-term HI predicts postnatal dystonic hypertonia (Derrick et al., 2009). Interestingly, in this rabbit model, motor deficits progress from initial hypotonia to hypertonia, mimicking human cerebral palsy but in a compressed timeframe (Rao et al., 2011). The presence of deep brain injury and a quantitative shift from hypo- to hypertonia has been associated with patients at risk for developing cerebral palsy. Although access to the rabbit model is limited, it could complement rodent studies in drug discovery, contributing important information on motor deficits.

#### *In vitro* models

Organotypic brain slice cultures are an invaluable intermediate between cell lines and *in vivo* models, because they largely maintain the structural complexity, synaptic organization and receptor expression of the tissue (Humpel, 2015). In addition, they preserve the interactions between neurons and glial cells, which support the energetic status of neurons in ischemic conditions. They can also be used as screening platforms to identify novel therapeutics. Hippocampal slice cultures, in particular, can be subjected to transient oxygen–glucose deprivation and subsequent reoxygenation to simulate ischemic neuronal injury and reperfusion (Li et al., 2016). In addition, these cultures respond to therapeutic hypothermia, thus allowing the testing of neuroprotective agents in combination with standard-of-care hypothermia (Carloni et al., 2018). Assessing the range of neuroprotective concentrations of a given agent *in vitro*, in conjunction with PK determination of plasma and cerebral spinal fluid levels, is important for defining the therapeutic dosage in *in vivo* models and, ultimately, in patients.

### Assessment of preclinical models and clinical application

Studies in newborn piglet, rodent and sheep encephalopathy models have been instrumental in the development of therapeutic hypothermia for term infants with HIE (Gunn and Thoresen, 2015). In addition to hypothermia, erythropoietin (EPO) has been evaluated in rodent and NHP models, supporting the safety and efficacy of multiple EPO doses for improving histological and functional outcomes after HI (van der Kooij et al., 2008; Traudt et al., 2013). Small clinical trials of EPO in neonates with HIE have provided evidence supporting safety and preliminary efficacy in humans (Razak and Hussain, 2019), confirming the predictive value of animal models.

The use of precise, carefully selected animal models can help improve strategies to protect babies with moderate to severe HEI. Nevertheless, there is no single animal model that can fully recapitulate the complexity of this condition. Thus, a proof of concept for efficacy of an emerging therapeutic strategy should be obtained in at least two different models before embarking on further clinical development. Experimental neuroprotective treatments should be initiated after completion of the insult to simulate a therapeutic intervention and should also be tested in combination with therapeutic hypothermia. Rodent models are the most cost effective in a drug discovery setting and offer genetic homogeneity. Nevertheless, large animals, such as piglets, are important not only to further evaluate therapeutic efficacy in gyrencephalic brains and to explore adjunct therapy, but also for more accurate PK and initial safety studies.

## NS

Sepsis is a systemic inflammatory response to infection. Such infections can be caused by Gram-negative or Gram-positive bacteria, fungi, parasites and viruses. It is accepted that sepsis consists of both a pro-inflammatory response at the onset of infection and an anti-inflammatory counter-response that occurs afterwards.

NS is categorized into early- and late-onset and develops rapidly. Early-onset NS presents within 7 days of life or during the first 72 h after birth in the case of VLBW preterm infants. It is associated with maternal acquisition via transplacental infection or via ascending infection from the cervix. The incidence is ∼1–2 per 1000 live newborns, with a mortality rate of ∼3% in term newborns (Table 1) and ∼16% in VLBW infants (Cortese et al., 2016). Group B *Streptococcus* (GBS) and Gram-negative enterobacteria (predominantly *E. coli*) account for most early-onset NS cases. Vaginal or rectal cultures of pregnant women at term may show GBS colonization rates of up to 35%, and intrapartum antibiotic prophylaxis administered to GBS-positive mothers has reduced the incidence of early-onset infection by 80% (Verani and Schrag, 2010).

Late-onset NS occurs at 4–90 days of life and is usually acquired from the caregiving environment. Preterm infants, especially VLBW infants, are most susceptible due to prolonged hospitalization, mechanical ventilation, intravascular catheters and immaturity of the immune system. The incidence is ∼25–30% in VLBW infants and ∼6–10% in late preterm newborns (Table 1). Staphylococci account for 30–60% of late-onset NS and most frequently infect via intravascular devices, particularly central vascular catheters. *E. coli* is also a significant cause of late-onset NS, especially in VLBW infants. More recently, several clinical cases have suggested that GBS transmission can also occur postpartum through breast milk. Treatment includes empiric broad-spectrum antibiotics until therapy can be tailored to target the specific pathogen.

### Preclinical models of NS

NS models are distinct from adult sepsis because adult animals rely on both adaptive and innate immunity for survival, while neonates rely more heavily on their innate immune responses. *In vivo* models of NS and its treatments have been reported in mouse, rat, rabbit, pig, humanized mouse and zebrafish, in which common pathogens, such as GBS, *E. coli* and *Staphylococcus aureus*, have been studied. Some of these better mimic human NS, while others do not fully reflect human pathophysiology, so interpretation of any results should take this into consideration (Cao and Robinson, 2014).

Zebrafish are easy to genetically manipulate, lay large clutches and are transparent for the first week of life. Additionally, the zebrafish and mammalian innate immune systems are strikingly similar. Examination of GBS infection in zebrafish larvae at 3 days postfertilization revealed susceptibility to GBS, and bacteria cross the blood–brain barrier (Kim et al., 2015). Furthermore, infection with virulence factor-deficient bacterial mutants is attenuated. These findings propose a system for examining GBS virulence, which allows for the visualization of infection *in vivo*. Subsequently, models utilizing LPS to induce endotoxemia and infection with *Edwardsiella tarda* to establish a zebrafish model of sepsis were reported (Philip et al., 2017; Wen et al., 2018). These zebrafish models exhibit many of the major hallmarks of human sepsis and show potential for high-throughput screening.

Cecal ligation and puncture (CLP) in mice (Table 4) is a widely used experimental model for adult sepsis (Ward, 2012). This model is considered the ‘gold standard’ because of its simple procedure and its ability to mimic human local and systemic responses in sepsis (Dejager et al., 2011). In neonatal mice, CLP is technically challenging due to their small size, gut friability and the increased risk of maternal cannibalization. Wynn et al. (2007) were unable to perform CLP in neonatal mice due to these technical challenges. CLP has also been associated with a large degree of variability between individual investigators because of the required size and number of enterotomies and the location of cecal ligation. An alternative to CLP is the cecal slurry model of generalized peritonitis (Table 4). In adult rats, this model recapitulates the time course and many of the physiological changes associated with human clinical sepsis, and has been adapted to induce polymicrobial sepsis in both adult and neonatal mice (Wynn et al., 2007). Young adult (7–10 weeks) and neonatal (5–7 days) mice received intraperitoneal administration of varying doses of a suspension of cecal contents from adult mice. Neonatal mice were more susceptible to sepsis and mounted a markedly attenuated systemic inflammatory response compared with young adult animals (Wynn et al., 2007). Brook et al. have more recently published methods and recommendations for the standardization of the neonatal mouse cecal slurry model (Brook et al., 2019a,b).

**Table 4.**
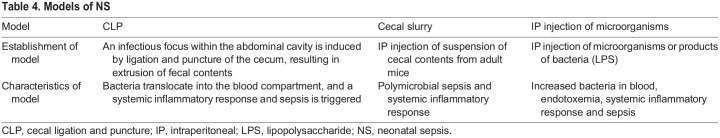
Models of NS

The immature immune system is primarily linked to the rapid disease progression in neonates. To mimic this naive immune system *in vivo*, some investigators have used humanized mice, wherein CD34^+^ hematopoietic stem cells from human cord blood are transplanted into immunodeficient animals. Some human immune cell populations, such as neutrophils, are present in low numbers or exhibit functional deficiencies in these mice, recapitulating the impaired human neonatal immune system. In addition, the protective environment of pathogen-free husbandry facilities maintains a naive adaptive immune cell phenotype that is similar to that of neonates. Another immunological similarity between neonates and humanized mice is complement deficiency, which may impair clearance of bacterial infections (Ernst et al., 2013).

Mouse and rat models of NS have also been generated by intraperitoneal pathogen injection. These include type III GBS strain (Rodewald et al., 1992; Seifert et al., 2006), a methicillin-resistant strain of *S. aureus*, which is the second most common pathogen for late-onset sepsis among VLBW infants (Placencia et al., 2009), and infection with a single strain of *Ureaplasma* (Weisman et al., 2012) (Table 4).

Neonatal rats possess the same age-related susceptibility to GBS as human neonates and have been used widely to model GBS infection and to evaluate the ability of the bacteria to disseminate systemically and survive significant exposure to host defense mechanisms. However, this model bypasses the early stages of the infection, including adherence to and colonization of epithelial surfaces; hence, this model fails to recapitulate bacterial factors important to these stages in pathogenesis (Jones et al., 2000). In addition to GBS, several investigators have used *E. coli* or LPS in rat models of NS (Bortolussi et al., 1978; Jones et al., 2000; Seepersaud et al., 2006; Venkatesh et al., 2007; Lemaitre et al., 2014).

Although CLP is technically challenging in neonatal mice, it can be performed in 3-day-old piglets. A piglet CLP model of generalized peritonitis has been developed, similar to the mouse model (Table 4). As previously mentioned, CLP is a widely used model for adult sepsis. In the piglet, CLP evoked a state of shock resulting in elevated oxidative stress and IL-6 levels, a promising candidate cytokine for the diagnosis of NS and a marker of the activation of the cytokine network (Goto et al., 2012). In another piglet model, septic shock is induced by intravenous injection of *E. coli* LPS, which is technically easier compared to CLP, as it does not require surgery (Fischer et al., 2009).

### Investigating NS *in vitro*

*In vitro* studies of NS most often investigate a disease pathway, immune response or therapeutic hypothesis – for example, the differences in signaling pathways between infection with GBS and *Streptococcus pnuemoniae* – with the goal of identifying targets for adjunctive therapy (Kenzel et al., 2006). An *in vitro* study of antibiotic treatment of blood samples from healthy and septicemic foals found that aminoglycoside antibiotics were less likely to induce endotoxemia, a dangerous inflammatory complication related to sepsis, compared to beta-lactam antimicrobials (Bentley et al., 2002). A similar study could be done on NS patient blood samples to inform the use of appropriate antibiotics. Further, transcytosis assays using human epithelial cell monolayers can evaluate the potential for *E. coli* and other NS pathogens to penetrate across the gut barrier into the blood stream (Huang et al., 2009). This assay could screen novel potential therapies for the most promising candidates to evaluate further in animal models.

### Assessment of preclinical models and clinical application

The most common preclinical models of NS involve CLP and the injection of bacteria or bacterial products. Although these models replicate the pathophysiology of human NS, the mediators and specific mechanisms do not always translate well. Mouse models are most often used due to the ease of use and availability of genetic models; however, differences in the murine and human response to sepsis at the genomic level is an issue (Lewis et al., 2016). Many therapeutic approaches for NS that have shown efficacy in rodent models have failed in clinical trials, suggesting that these preclinical models do not effectively recapitulate the human syndrome. Criticism has been primarily focused on NS models produced by the intravenous administration of live bacteria or microbial products. Bacterial injection-based models may be useful, but differ from the human situation. Human neonates experience a relatively constant exposure to bacteria, whereas animals receive bolus doses. This can lead to discrepancies, for example, the therapeutic promise of TNF-α inhibitors in the LPS murine model of NS, but not in the CLP model. As TNF-α inhibitors offer very limited therapeutic benefit in the clinic, the LPS model is more predictive of the human response (Lewis et al., 2016). The improved predictive value of the CLP model may be due to its complexity and physiological relevance. Although some NS models show relevance and promise, predicting the human response to potential therapies remains challenging due to the complex multi-organ nature of sepsis.

## Conclusions

Through a review of the literature, we have identified and evaluated the available preclinical models for five common neonatal conditions with unmet clinical needs. Although the conditions are diverse and require specific approaches, several high-level, overarching key lessons can be learned by assessing the strengths, weaknesses and gaps in the available models.

Rodent models are most commonly used due to the ease of experimentation and facility requirements, relatively low cost and the availability of genetic models. However, the shortcomings and limitations of rodent models should be considered when translating results. Sheep, pig and NHPs appear to be most useful for some of the neonatal diseases, but are not widely used, likely due to the challenges and cost of husbandry and maintenance. Although practical aspects of the research program should be considered (e.g. species selection), this must be balanced with doing the best science to appropriately answer a research question. ROP and NEC research has demonstrated that the premature pig very closely mimics critical aspects of these diseases due, in part, to the ability to work with premature animals. Therefore, pigs should be considered over other models, despite the associated technical challenges.

A common element across BPD, ROP, NEC and NS is the overactivation of the innate immune system, which results in development or exacerbation of disease. Yet, this is not well captured in the available preclinical models and is an area of focus for future model development and improvement. Further understanding of species-specific immune system development and innate immune responses will aid in model development. Another gap, as described for BPD and NEC, is that improved knowledge and understanding of the clinical phenotype and pathogenesis is needed to inform preclinical model development and utility.

In many cases, treatments that are effective in models do not translate to clinical efficacy, which can be reflective of inherent species and developmental differences. Other issues may include the lack of standardized protocols and/or lack of understanding of the underlying disease mechanisms. We suspect that inefficient clinical translation is not only due to the difficulty of assessing neonate patients, but is rather due to issues with animal-to-human translation that are common in drug development. These are exacerbated in neonates due to the less robust characterization of the clinical disease and the highly dynamic period of morphologic and functional organ development that occurs at different times and rates across species.

For any specific disease, no single animal model completely covers the complexity of the human condition. It is recommended that a potential therapy is evaluated in multiple models to cover all aspects of a particular disease and to gain further confidence prior to advancing therapeutic agents into clinical evaluation. Although no ‘perfect’ animal model exists, preclinical models are useful and can be informative when judiciously applied. There are important points to consider when selecting an existing model or developing a new one to answer a specific translational question. These include the physiological similarities and differences between species at the neonate stage, recognition of the strengths and limitations of each model, the biological mechanisms underlying the disease phenotype and the specific translational research focus. Researchers should consider areas in which models such as zebrafish, *in vitro* human-derived systems and *in silico* approaches could help fill in the gaps and become part of a battery of tests to facilitate an integrated and holistic translational research program while limiting animal use, consistent with the 3Rs. Such integration is essential to ensure successful development of therapeutics for these neonatal diseases with high unmet medical needs.
